# Systematic approach to identify therapeutic targets and functional pathways for the cervical cancer

**DOI:** 10.1186/s43141-023-00469-x

**Published:** 2023-02-01

**Authors:** Md. Tanvir Hasan, Md. Rakibul Islam, Md. Rezwan Islam, Baraa Riyadh Altahan, Kawsar Ahmed, Francis M. Bui, Sami Azam, Mohammad Ali Moni

**Affiliations:** 1 Department of Business Engineering, Ghent University, 9000 Gent, Belgium; 2 Department of Software Engineering, Daffodil International University (DIU), Ashulia, Savar, Dhaka, 1342 Bangladesh; 3 Department of Medical Instrumentation Engineering Techniques, Al-Mustaqbal University College, Hilla, Babil 51001 Iraq; 4 Department of Electrical and Computer Engineering, University of Saskatchewan, 57 Campus Drive, Saskatoon, SK S7N 5A9 Canada; 5 Group of Bio-photomatiχ, Department of Information and Communication Technology, Mawlana Bhashani Science and Technology University (MBSTU), Santosh, Tangail, 1902 Bangladesh; 6 College of Engineering, IT and Environment, Charles Darwin University, Casuarina, NT 0909 Australia; 7 Faculty of Health and Behavioural Sciences, School of Health and Rehabilitation Sciences, The University of Queensland, St. Lucia, QLD 4072 Australia

## Abstract

**Background:**

In today’s society, cancer has become a big concern. The most common cancers in women are breast cancer (BC), endometrial cancer (EC), ovarian cancer (OC), and cervical cancer (CC). CC is a type of cervix cancer that is the fourth most common cancer in women and the fourth major cause of death.

**Results:**

This research uses a network approach to discover genetic connections, functional enrichment, pathways analysis, microRNAs transcription factors (miRNA-TF) co-regulatory network, gene-disease associations, and therapeutic targets for CC. Three datasets from the NCBI’s GEO collection were considered for this investigation. Then, using a comparison approach between the datasets, 315 common DEGs were discovered. The PPI network was built using a variety of combinatorial statistical approaches and bioinformatics tools, and the PPI network was then utilized to identify hub genes and critical modules.

**Conclusion:**

Furthermore, we discovered that CC has specific similar links with the progression of different tumors using Gene Ontology terminology and pathway analysis. Transcription factors-gene linkages, gene-disease correlations, and the miRNA-TF co-regulatory network were revealed to have functional enrichments. We believe the candidate drugs identified in this study could be effective for advanced CC treatment.

## Background

In 2020, an estimated 19.3 million new cancer cases (18.1 million excluding nonmelanoma skin cancer) were diagnosed worldwide, with around 10.0 million cancer deaths (9.9 million excluding nonmelanoma skin cancer) [1]. The global cancer burden is expected to be 28.4 million cases in 2040, a 47% increase from 2020, with a greater increase in transitioning (64 to 95%) countries versus transitioned (32 to 56%) countries due to demographic changes, though this may be exacerbated further by increasing risk factors associated with globalization and a growing economy [2, 3]. Lung cancer and colorectal cancer are the most prevalent cancers in men, whereas breast cancer, colorectal cancer, cervical cancer, and lung cancer are the most common in females [4–6]. In 2020, men had a 19% higher overall cancer incidence rate (222.0 per 100,000) than women (186 per 100,000), while rates varied greatly across areas. Cervical cancer is the fourth most common malignancy in women and the fourth greatest cause of cancer mortality, with an estimated 604,000 new cases and 342,000 deaths in 2020 [7]. The major risk factor for CC is infection with particular forms of HPV, followed by smoking [1, 8]. Types of HPV are 16 and 18 accounting for 75% of all CC instances globally, with HPV types 31 and 45 accounting for the remaining 10% [9]. There are approximately 150–200 different types of HPV that have been identified, with 15 being considered high risk (16, 18, 31, 33, 35, 39, 45, 51, 52, 56, 58, 59, 68, 73, and 82), three being considered probable high risk (26, 53, and 66), and 12 being considered low risk (6, 11, 40, 42, 43, 44, 54, 61, 70, 72, 81, and CP6108) [10].

An investigation of differentially expressed gene (DEG) promoter sequences and transcription factor (TF) binding sites previously identified TF E2F as a crucial transcriptional regulator and a possible molecular target for cervical cancer treatment [11]. Another research found that the target genes CDC45, GINS2, MCM2, and PCNA are important participants of cervical cancer [12]. Several novel candidate genes implicated in cervical carcinogenesis (e.g., VEGFA and IL-6) were also predicted by combining human protein interaction data with cervical cancer gene sets [13, 14]. These researches have provided important information concerning cervical cancer, but no conclusions about the disease’s underlying molecular pathways were made. Another wisely analyzed study has demonstrated that the predictive potential reporter biomolecules including KAT2B, PCNA, CD86 [15, 16], miR-192-5p, and miR-215-5p have a significant role in cervical cancer [17].

Three datasets have been taken to complete this network-based study. The common DEGs were then determined by comparing the datasets. Using common DEGs have applied for GO annotation, pathway analysis, construct PPI network, module analysis, hub DEGs identification, gene-disease association prediction, TF-miRNA co-regulatory network identification, and drug target prediction.

## Methods

**Fig. 1:**
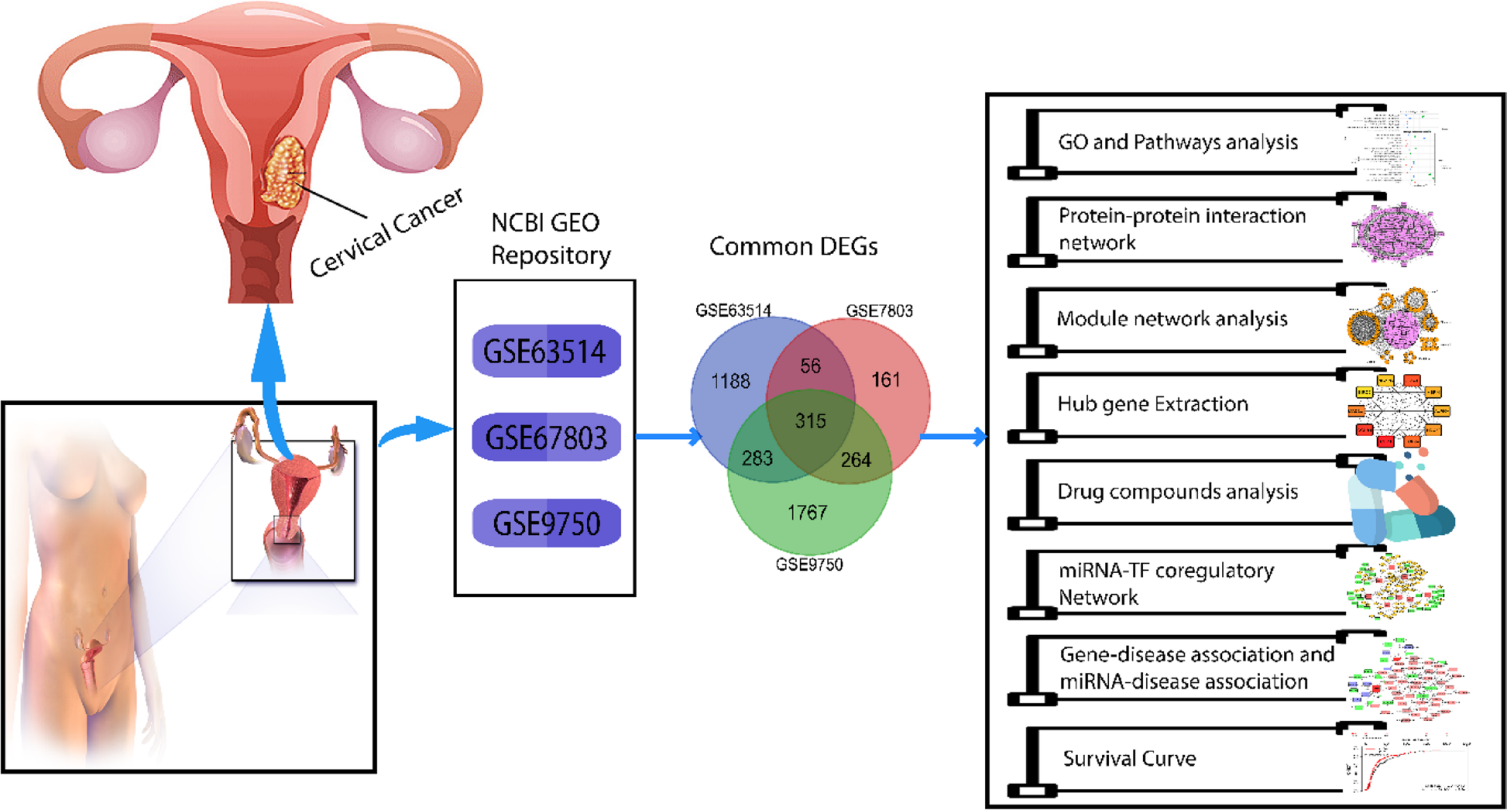
Image workflow of this study methods. A transcriptomic comparative analysis was performed for CC. Three datasets were gathered for CC, and GEO2R was used to detect differentially expressed genes (DEGs), and the datasets were filtered to normalize and find common DEGs. Using shared datasets has performed various transcriptomic analyses

The study effort has included differential expressed genes (DEGs) discovery, common DEGs finding, gene ontology analysis, pathways enrichment analysis, protein interaction network building, module analysis, TF-miRNA co-regulatory network, drug compounds analysis, and gene-disease correlations network creation, which is a network-based approach. All methodology phases are detailed here, and a quick representation of this work is demonstrated in Fig. 1.

### Data collection

The GEO database is a free public resource that stores and distributes high-throughput gene expression data from all over the world (https://www.ncbi.nlm.nih.gov/geo/) [18–20]. Three datasets have been considered for CC for this study including GSE9750, GSE63514, and GSE7803 from the GEO repository. GSE9750 stands on a single platform GPL96 [HG-U133A] Affymetrix Human Genome U133A Array for *Homo sapiens* organisms named “Identification of gene expression profiles in CC.” This dataset conducts total of 66 samples where 42 samples are affected and 24 samples are normal cervical cell [21]. GSE63514 dataset depends on a single platform GPL570 [HG-U133_Plus_2] Affymetrix Human Genome U133 Plus 2.0 Array. This dataset conducts a total of 128 samples where 104 are affected cells and 24 normal cells [22]. And the last dataset GSE7803 conducts 10 normal cells and 31 affected cells [23].

### DEGs identification

GEO2R was utilized to find the DEGs. The raw file was transformed to expression using a statistical relevance *p*-value of 0.05 and logFc > 1 (upregulated) and logFc-1 (downregulated), and the common DEGs were identified using a Venn diagram web tool (http://bioinformatics.psb.ugent.be/webtools/Venn/) [24].

### Ontological terms and pathways analysis

The biological activities of the common DEGs were identified via gene ontology analysis using the Gene Ontology database [25], while the corresponding pathways were discovered in the KEGG database [26]. The Enrichr online tool [27] was used for all of the analyses. Enrichr is a web-based intuitive enrichment analysis application that provides many sorts of graphical summaries of gene list collective functions. Enrichr is an open-source project that can be found at http://amp.pharm.mssm.edu/Enrichr [28].

### Construction of PPI network and clustering analysis

The PPI network [29] is a graphical representation of gene interaction. The PPI network was built using the physical interactions of proteins from frequent DEGs in CC datasets from the STRING database [30]. To predict the interconnection between the proteins was set the minimum confidence score of 0.40. Afterward, the open-source Cytoscape tool was used to analyze the PPI network [31]. To identify the complex network part (cluster) of the PPI network, the MCODE method was implemented [32]. The MCODE basic parameters degree cutoff = 2, node score cutoff = 0.2, k-core = 2, and maximum depth = 100 were chosen as a minimum criterion. Using the connection (degree) technique, the cytoHubba plugin program of Cytoscape software was used to discover hub DEGs from the PPI network [33].

### Drug target analysis

The drug signatures database (DSigDB) drug signature database [34] was used to identify therapeutic targets for selected hub DEGs. The DSigDB is a new gene set resource for gene set enrichment analysis that links drugs/compounds to their target genes (GSEA). DSigDB now has 22,527 gene sets, each of which contains 17,389 unique compounds spanning 19,531 genes. Users may search, browse, and download drugs, chemicals, and gene sets from the DSigDB database. In GSEA software, DSigDB gene sets may be utilized to connect gene expression to drugs/compounds for drug repurposing and translational research [34]. The cutoff criterion for identifying pharmacological targets was *p*-value 0.01 and overlapping genes count > = 9.

### Association network of gene disease

Linkage studies [35], genome-wide association studies (GWAS) [36], and RNA interference screens [37] are all expensive and time-consuming approaches for determining gene-disease associations. As a result, a variety of computational methods [38–40] for identifying or predicting gene-disease correlations have been developed. These approaches have distinct strengths and limitations, and they are best suited to different types of diseases [41]. Gene-disease association network has been exported through DisGeNET database (https://www.disgenet.org/).

### TF-miRNA co-regulatory network development and analysis

The RegNetwork repository (http://www.regnetworkweb.org/) was used to build the TF-miRNA co-regulatory networks that revealed new information about the key role of proposed TF-miRNA co-regulation in CC and its crosstalk with the surrounding microenvironment [42]. RegNetwork is a human and mouse gene regulatory network repository that collects and combines known regulatory connections between TFs, miRNAs, and target genes from 25 different datasets. RegNetwork is a database that houses a comprehensive collection of empirically known or predicted transcriptional and posttranscriptional regulatory interactions, and the database structure is flexible enough to accommodate future additions into gene regulatory networks for more species [43].

### Analysis of survival for hub genes

A time-to-event study, also known as a survival analysis, refers to a set of approaches for long-term research prior to the identification of a specific endpoint of interest. The occurrence (e.g., death) does not frequently occur in all patients at the end of the observation period [44–46], which is a distinctive feature of the survival data. In this analysis, the survival role was calculated for altered and regular classes of significant genes common to datasets of CC by using Cox PH and PL estimators. The Cox PH regression model was evaluated univariate as well as multivariate [47].

## Results

### Three-hundred fifteen common DEGs identified

**Fig. 2:**
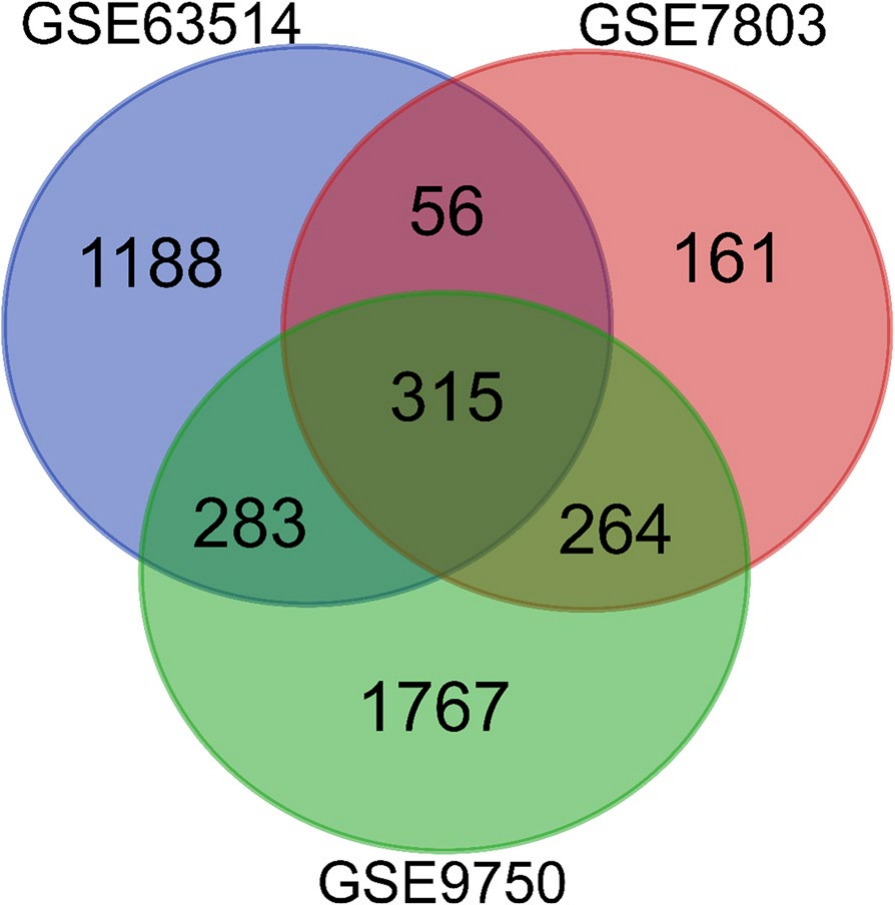
A comparison method was applied between the datasets including GSE63514, GSE7803, and GSE9750 to identify the common DEGs. A total of 315 common DEGs were identified for further analysis

Using GEO2R for CC, the dataset was downloaded from the GEO repository of NCBI. Initially, the GSE9750, GSE63514, and GSE7803 datasets revealed 21,156, 45,118, and 20,056 DEGs, respectively, after filtration with the criteria 2629, 1842, and 796 DEGs were taken. Following that, a comparison of the DEGs revealed 315 shared DEGs (Fig. 2).

### Ontological terms and pathways analysis

**Fig. 3:**
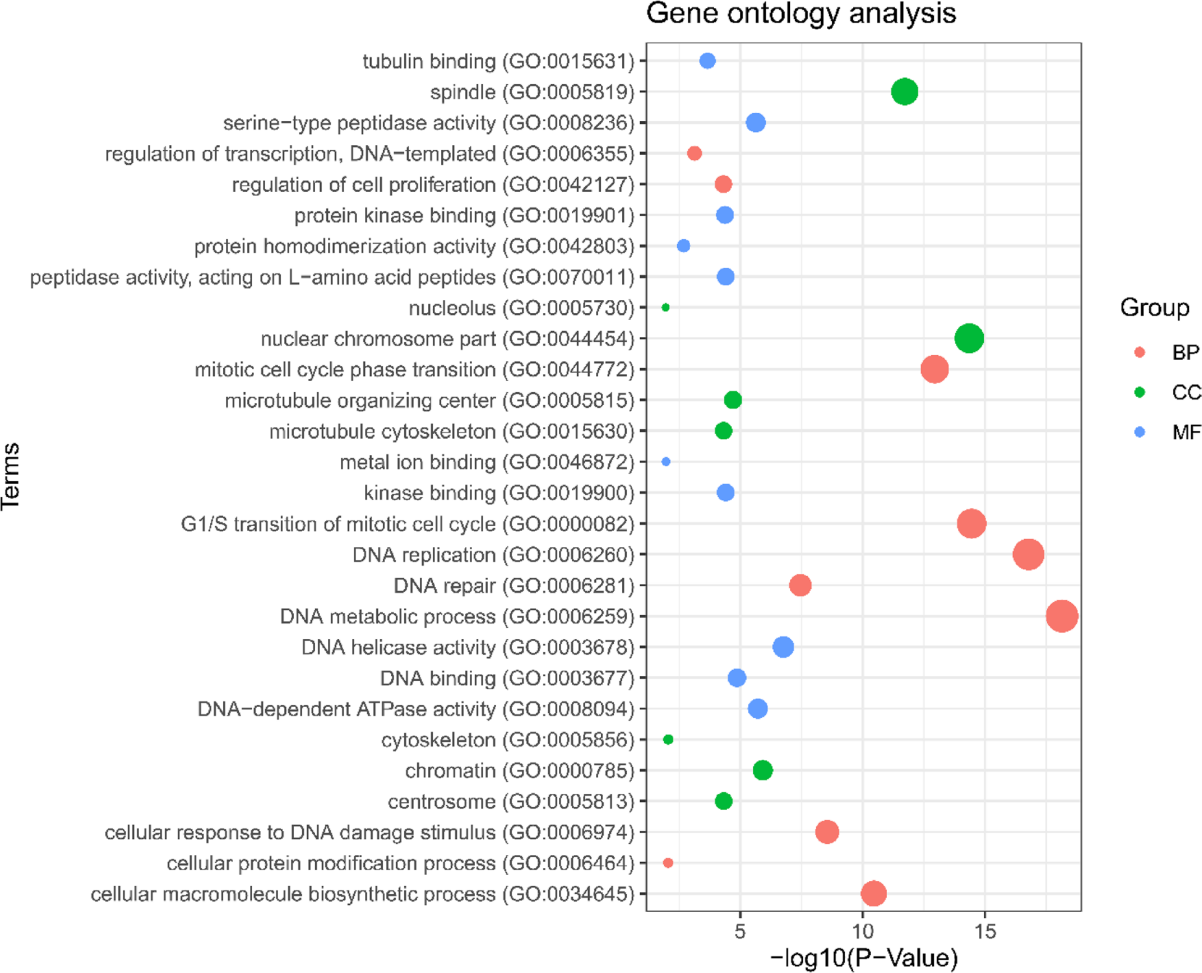
Gene ontological analysis for BP, CC, and MF. Red-colored terms indicate BP, green-colored terms indicate CC, and blue-colored terms indicate MF. According to figure, DNA metabolic process (GO: 0006259) is the most significant term from the BP ontology

**Fig. 4:**
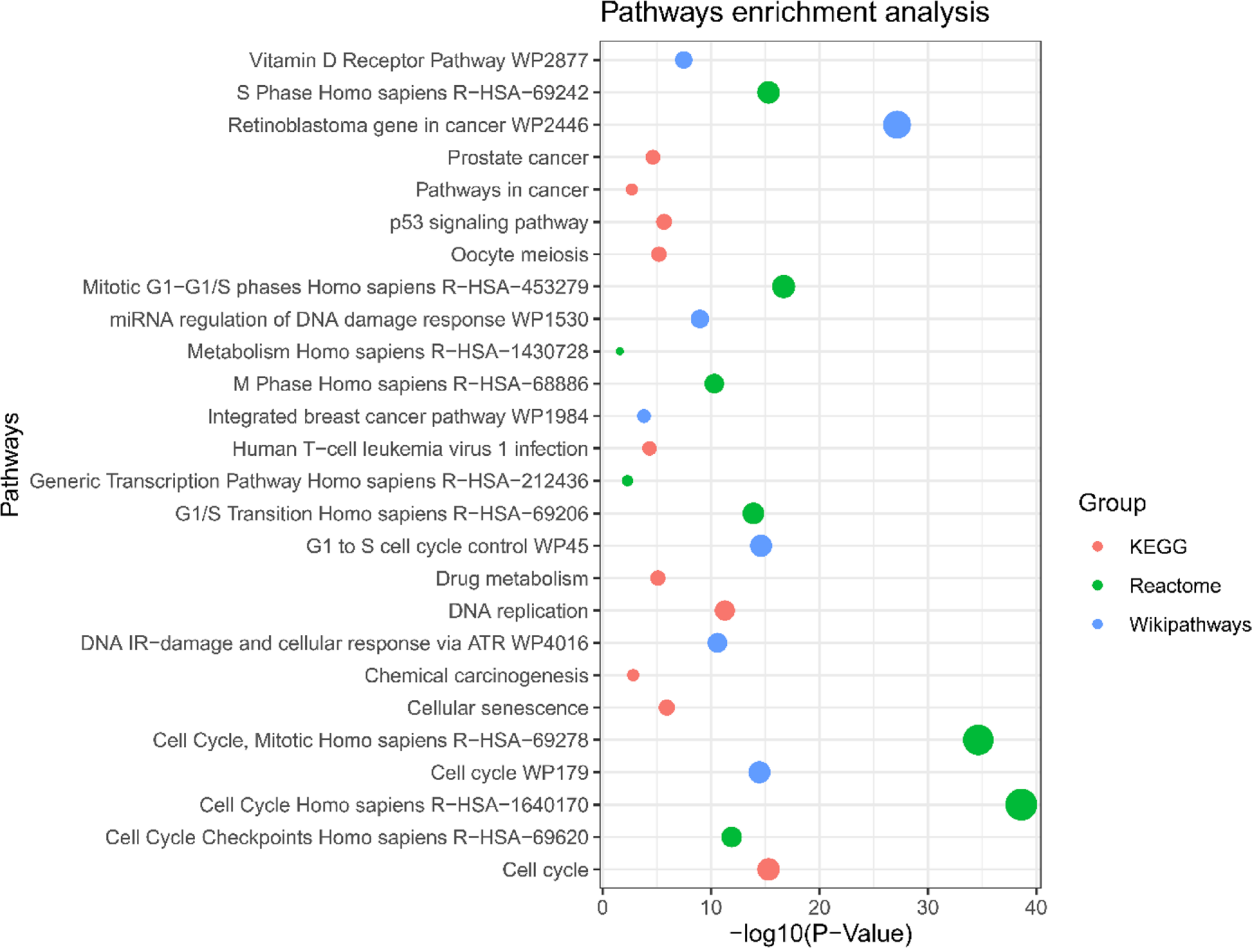
Pathways enrichment analysis through three databases including KEGG, Reactome, and WikiPathways. All the revealed pathways had been performed *p*-value less than 0.05

The GO analysis reveals most of the DEGs associated with the terms including the following: DNA metabolic process, DNA replication, G1/S transition of mitotic cell cycle, mitotic cell cycle phase transition, cellular macromolecule biosynthetic process, cellular response to DNA damage stimulus, DNA repair, regulation of cell proliferation, regulation of transcription, DNA templated, cellular protein modification process for biological process, nuclear chromosome part, spindle, chromatin, microtubule-organizing center, centrosome, microtubule cytoskeleton, cytoskeleton, nucleolus for cellular component, DNA helicase activity, DNA-dependent ATPase activity, serine-type peptidase activity, DNA binding, peptidase activity, acting on L-amino acid peptides, kinase binding, protein kinase binding, tubulin binding, protein homodimerization activity, metal ion binding for molecular function, etc. (Table 1) (Fig. 3). The pathway of KEGG, Reactome, and WikiPathways analysis reveals that most of the common DEGs were associated with the cell cycle, DNA replication, cellular senescence, cell cycle, p53 signaling pathway, pathways in cancer, mitotic, mitotic G1-G1/S phases, S phase, M phase, generic transcription pathway, prostate cancer, metabolism, retinoblastoma gene in cancer, G1 to S cell cycle control, integrated breast cancer pathway, etc. (Table 2) (Fig. 4).

**Table 1:** Significant GO terms with their GO ID, overlapped genes, and *p*-value of the common DEGs associated with CC. In this table, BP means biological process, MF means molecular function, and CC means cellular component. All the GO terms which have been taken were filtered with a *p*-value less than 0.05. Basically, the *p*-value helps to determine the significance of the results in relation to the null hypothesis

Terms	GO ID	*p*-value	Overlapped genes	Group
DNA metabolic process	GO:0006259	7.16E-19	34	BP
DNA replication	GO:0006260	1.65E-17	22	BP
G1/S transition of mitotic cell cycle	GO:0000082	3.52E-15	19	BP
Mitotic cell cycle phase transition	GO:0044772	1.13E-13	24	BP
Cellular macromolecule biosynthetic process	GO:0034645	3.48E-11	27	BP
Cellular response to DNA damage stimulus	GO:0006974	2.79E-09	23	BP
DNA repair	GO:0006281	3.47E-08	20	BP
Regulation of cell proliferation	GO:0042127	4.85E-05	27	BP
Regulation of transcription, DNA templated	GO:0006355	7.29E-04	42	BP
Cellular protein modification process	GO:0006464	0.008786	26	BP
Nuclear chromosome part	GO:0044454	4.36E-15	33	CC
Spindle	GO:0005819	1.90E-12	21	CC
Chromatin	GO:0000785	1.19E-06	18	CC
Microtubule-organizing center	GO:0005815	1.96E-05	22	CC
Centrosome	GO:0005813	4.70E-05	20	CC
Microtubule cytoskeleton	GO:0015630	4.78E-05	18	CC
Cytoskeleton	GO:0005856	0.008643	16	CC
Nucleolus	GO:0005730	0.011101	19	CC
Serine-type peptidase activity	GO:0008236	2.30E-06	15	MF
DNA binding	GO:0003677	1.36E-05	32	MF
Peptidase activity, acting on L-amino acid peptides	GO:0070011	3.89E-05	12	MF
Kinase binding	GO:0019900	3.90E-05	19	MF
Protein kinase binding	GO:0019901	4.20E-05	21	MF
Tubulin binding	GO:0015631	2.16E-04	13	MF

**Table 2:** Pathways enrichment analysis with their *p*-value and overlapped genes of common DEGs for CC through three databases including KEGG, Reactome, and WikiPathways. The pathways of the cell cycle are the most significant which have been reported by the three databases

Pathways	***p***-value	Overlapped genes	Database
Cell cycle	4.92E-16	21	KEGG
DNA replication	5.26E-12	11	KEGG
Cellular senescence	1.19E-06	13	KEGG
p53 signaling pathway	2.13E-06	9	KEGG
Oocyte meiosis	6.35E-06	11	KEGG
Drug metabolism	8.07E-06	10	KEGG
Prostate cancer	2.26E-05	9	KEGG
Human T-cell leukemia virus 1 infection	4.70E-05	13	KEGG
Chemical carcinogenesis	0.001498	11	KEGG
Pathways in cancer	0.002009	18	KEGG
Cell cycle	2.40E-39	67	Reactome
Cell cycle, mitotic	2.20E-35	58	Reactome
Mitotic G1-G1/S phases	1.97E-17	23	Reactome
S phase	4.92E-16	21	Reactome
G1/S transition	1.22E-14	19	Reactome
Cell cycle checkpoints	1.24E-12	21	Reactome
M phase	4.93E-11	23	Reactome
Generic transcription pathway	0.005002	23	Reactome
Metabolism	0.025365	41	Reactome
Retinoblastoma gene in cancer	6.87E-28	27	WikiPathways
G1 to S cell cycle control	2.42E-15	16	WikiPathways
Cell cycle	3.43E-15	20	WikiPathways
DNA IR-damage and cellular response via ATR	2.57E-11	14	WikiPathways
miRNA regulation of DNA damage response	1.05E-09	12	WikiPathways
Vitamin D receptor pathway	3.27E-08	16	WikiPathways
Integrated breast cancer pathway	1.52E-04	10	WikiPathways

### Hub genes identification and clustering analysis

**Fig. 5:**
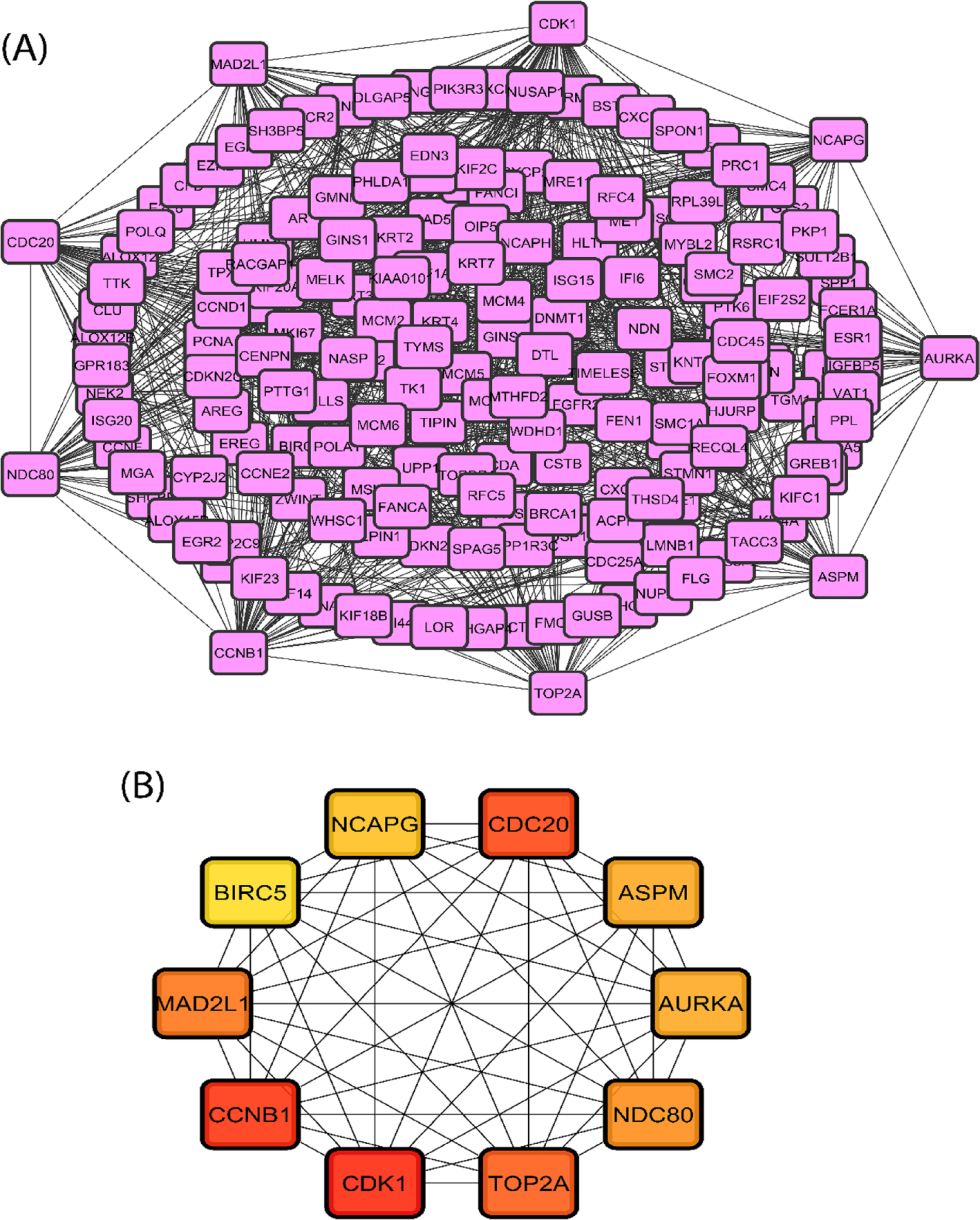
**A** Protein interaction for the common differentially expressed genes for cervical cancer. The network conducts 183 nodes (indicating genes) and 1082 edges (indicating connections between nodes). **B** The hub genes were identified from the protein interaction network. Top 10 significant genes were CDK1, CCNB1, CDC20, TOP2A, MAD2L1, NDC80, AURKA, ASPM, NCAPG, and BIRC5

**Fig. 6:**
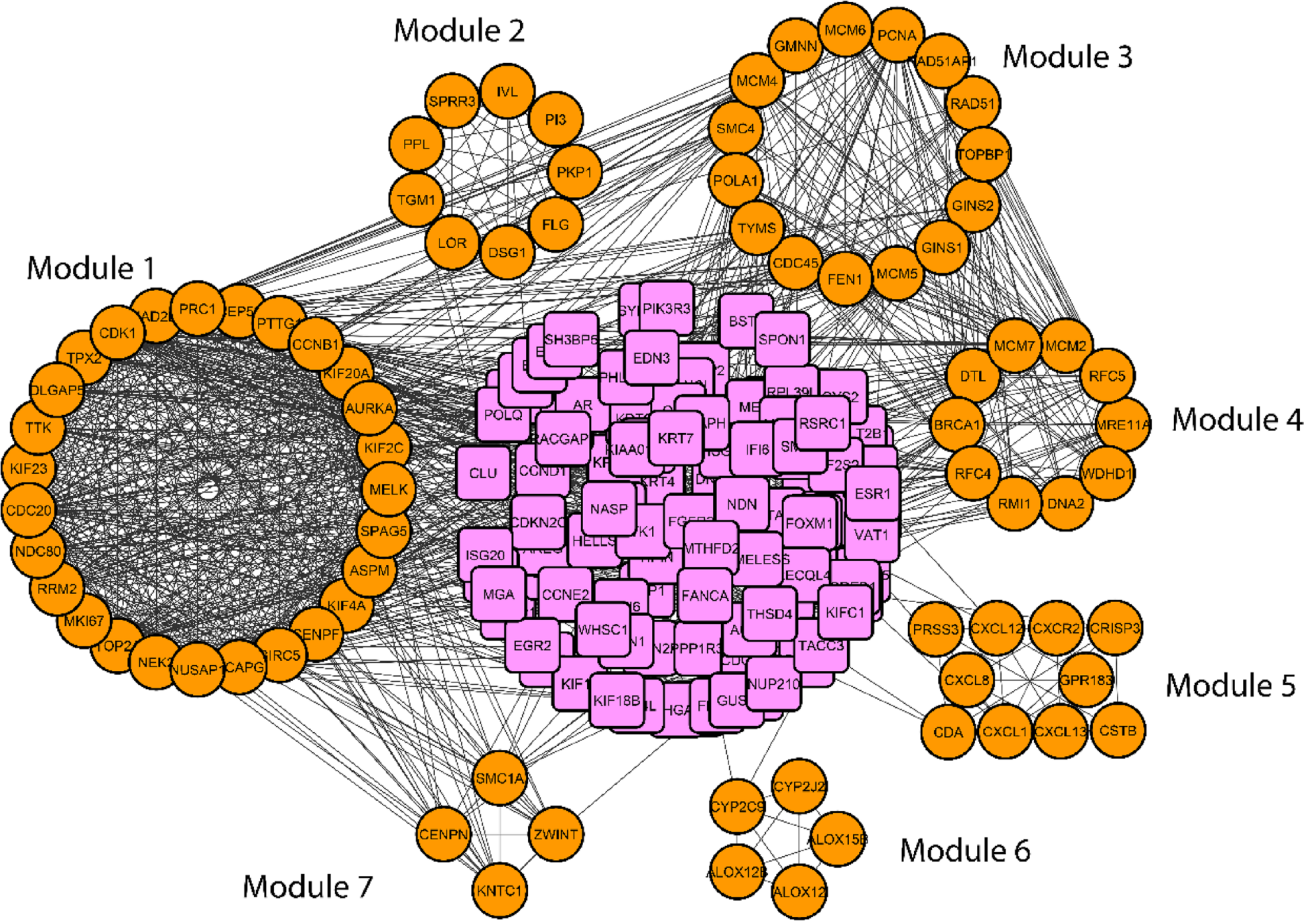
Seven significant modules could be extracted through the MCODE plugin tools of Cytoscape. The module is the very complex area of PPI network. In the figure, yellow color circular nodes represent the module network. There were seven module networks which have been obtained

The PPI network is a significant product of this sort of research. The STRING database was used to create the PPI network, which was then shown using the Cytoscape application. The PPI network (Fig. 5A) has 183 nodes and 1082 edges, with nodes representing DEGs and edges representing connections between nodes. Afterward, we have gotten 10 hub DEGs (CDK1, CCNB1, CDC20, TOP2A, MAD2L1, NDC80, AURKA, ASPM, NCAPG, and BIRC5) (Fig. 5B) using cytoHubba plugin tool of Cytoscape. By contrast, the MCODE plugin algorithm showed 10 modules (cluster sub-networks); from them, significant 7 modules have taken (Fig. 6). The first module carried 27 nodes and 316 edges, the second module contains 9 nodes and 36 edges, the third module conducts 15 nodes and 51 edges, and fourth modules carried 10 nodes and 26 edges.

### Lucanthone and paclitaxel target significantly interacted with the hub genes

The therapeutic target for the hub DEGs was discovered using the DSigDB drug target database. The result showed that the “LUCANTHONE CTD 00006227 and paclitaxel CTD 00007144” both of drug targets mostly associated with the hub DEGs (Table 3). Lucanthone is being studied as a chemotherapy and radiation sensitizer due to its potential to interact with DNA repair [48]. However, because lucanthone reduces cancer cell survival regardless of p53 status, autophagy suppression may be a more important contribution to the lucanthone mode of action that impacts DNA repair. Because lucanthone suppresses autophagy, it may be able to boost the effectiveness of chemotherapeutics that activate this process. Both apoptosis and autophagy have been observed to be induced by vorinostat and bortezomib, and inhibiting autophagy increases its activity [49–51]. Tumor immunotherapy improves the body’s immunity, resulting in the immunological response to malignancies. Our understanding of the potential utility of conventional medicines in tumor immunotherapy has advanced recently [52]. According to multiple studies, paclitaxel directly eliminates tumor cells while also regulating immune cells such as effector T cells, dendritic cells (DCS), natural killer (NK) cells, regulatory T cells (Tregs), and macrophages [53]. Belinostat [54], doxorubicin [55], bleomycin [56], and bortezomib [57] are examples of chemotherapeutics with comparable immunomodulatory characteristics. Paclitaxel is a novel anticancer medication having broad-spectrum action in epithelial ovarian cancer, head and neck cancer, esophageal cancer, breast cancer, CC, and lung cancer [58–60]. The Food and Drug Administration (FDA) has approved cisplatin and topotecan for the treatment of advanced cervical cancer. However, the cisplatin/paclitaxel or carboplatin/paclitaxel regimens are less toxic and easier to administer than cisplatin/topotecan according to National Comprehensive Cancer Network (NCCN) Guidelines Insights: Cervical Cancer, Version 1.2020 [61, 62]. The panel decided to add carboplatin/paclitaxel/bevacizumab to the list of recommended regimens for recurrent or metastatic cervical cancer based on the findings of GOG 240 and JGOG0505. According to prior research, cisplatin/paclitaxel and carboplatin/paclitaxel have become the most frequently utilized systemic regimens for metastatic or recurrent cervical cancer [61].

**Table 3:** Significant drug compounds with overlapped DEGs and *p*-value for hub DEGs. From the analysis of drug compounds, lucanthone and paclitaxel showed the high significance for the CC. Lucanthone and paclitaxel overlapped 10 and 9 hub DEGs respectively

Drug compounds	Overlapped genes	*p*-value
LUCANTHONE CTD 00006227	10	1.52E-20
Paclitaxel CTD 00007144	9	3.06E-14
Dasatinib CTD 00004330	9	3.60E-14
Troglitazone CTD 00002415	9	3.77E-13
Resveratrol MCF7 DOWN	6	3.53E-12
Dasatinib CTD 00004330	9	3.60E-14

### Gene-disease association

**Fig. 7:**
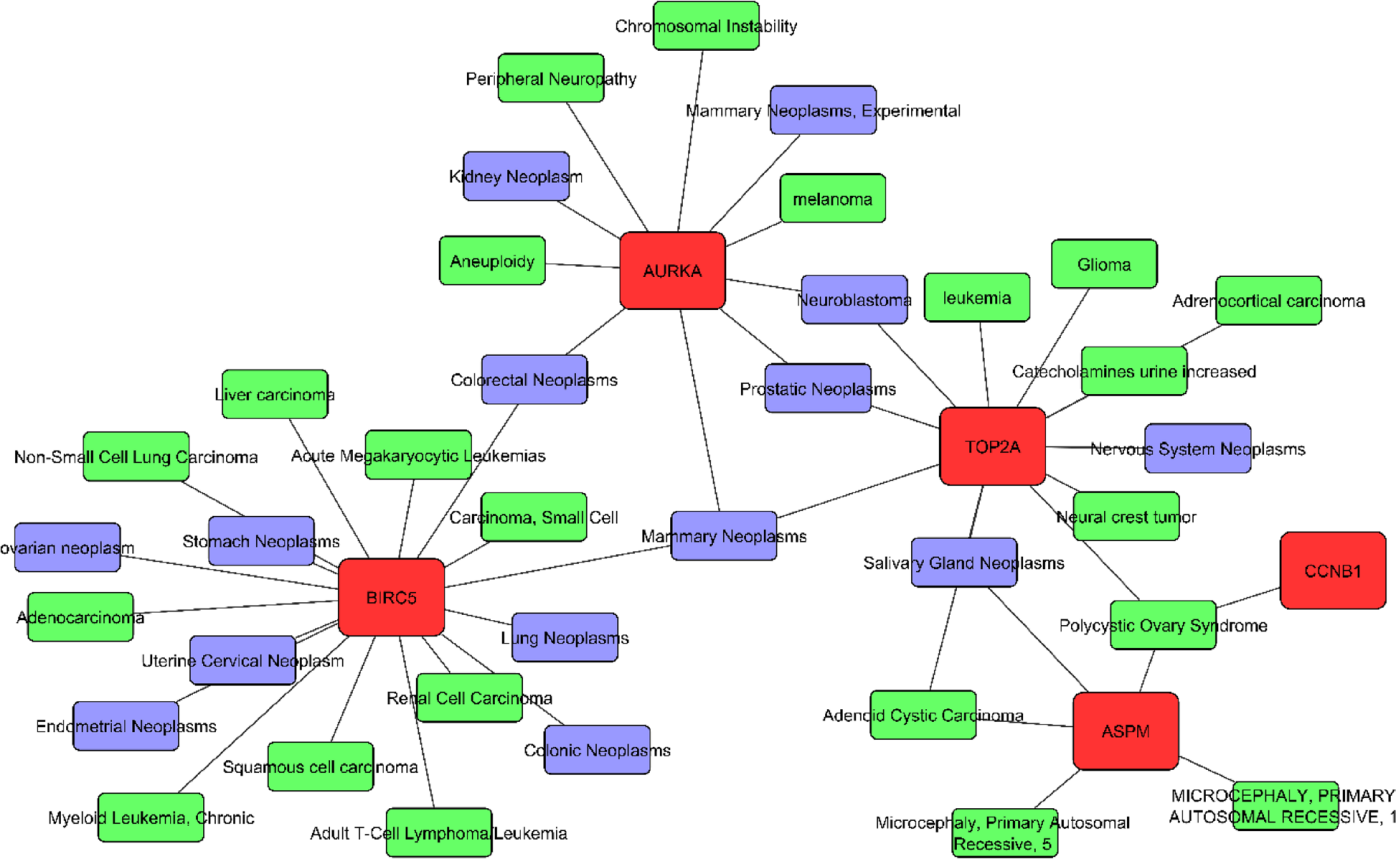
The hub DEGs linked diseases as illustrated by the gene-disease association network. Red nodes, hub genes; green, cancer-related genes; blue, neoplasms

Hub genes cooperated to explore the gene-disease interaction network through the DisGeNET database. In the gene-disease interaction, network genes and diseases are interconnected. A total of 10 hub genes were used to apply for the gene-disease association network where just 5 hub genes show the connectivity between the genes and diseases (Fig. 7). The network showed the BIRC5 gene is the most connected node that is associated with 17 cancers and tumor-related diseases.

### TF-miRNA co-regulatory network development and analysis

**Fig. 8:**
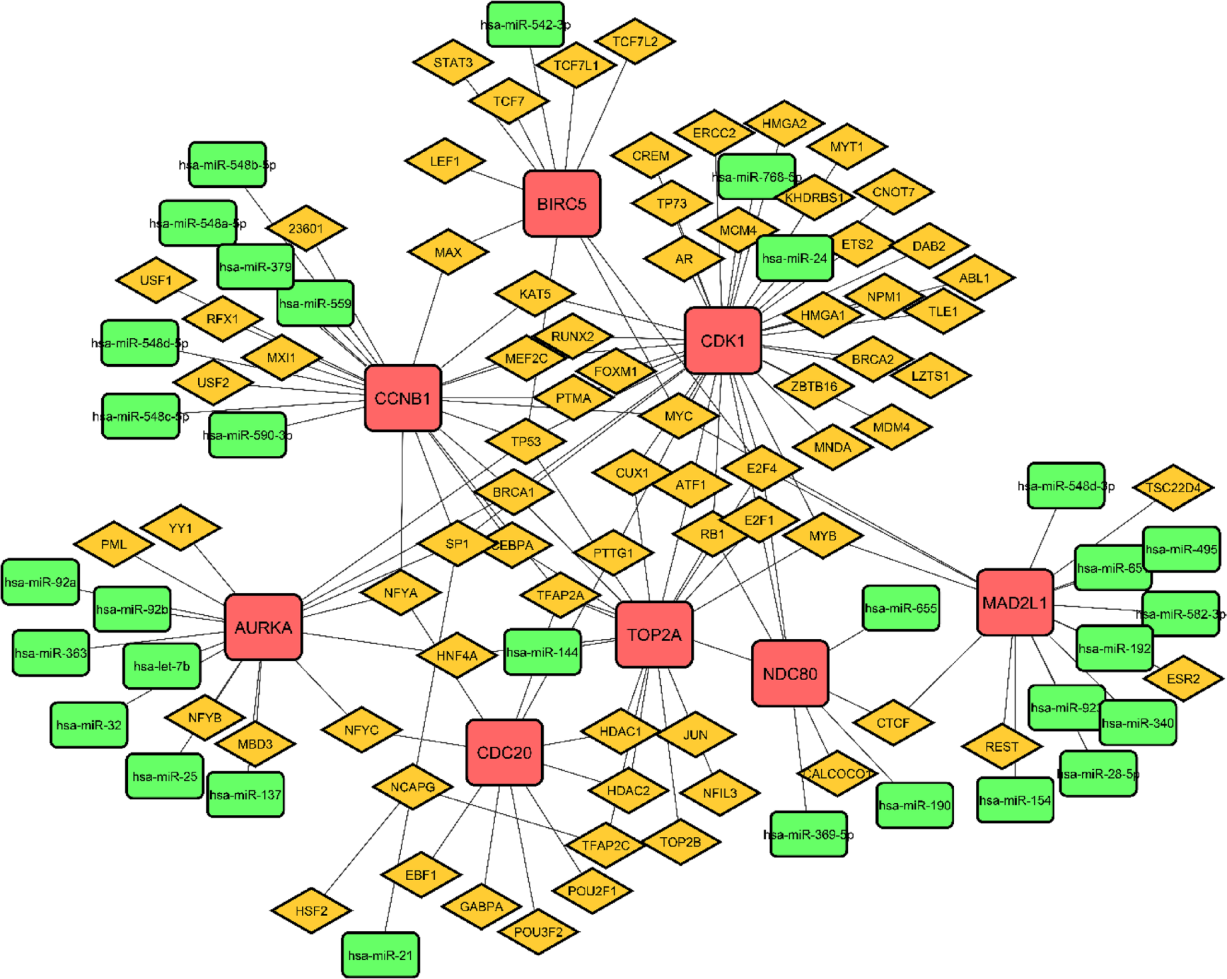
In this network, the TF genes and miRNA are interconnected named TF-miRNA co-regulatory network. Herein, the square shape red color nodes indicate hub DEGs, yellow color TF candidates shown as a diamond shape nodes, and rectangular green color nodes indicate miRNA candidates

**Fig. 9:**
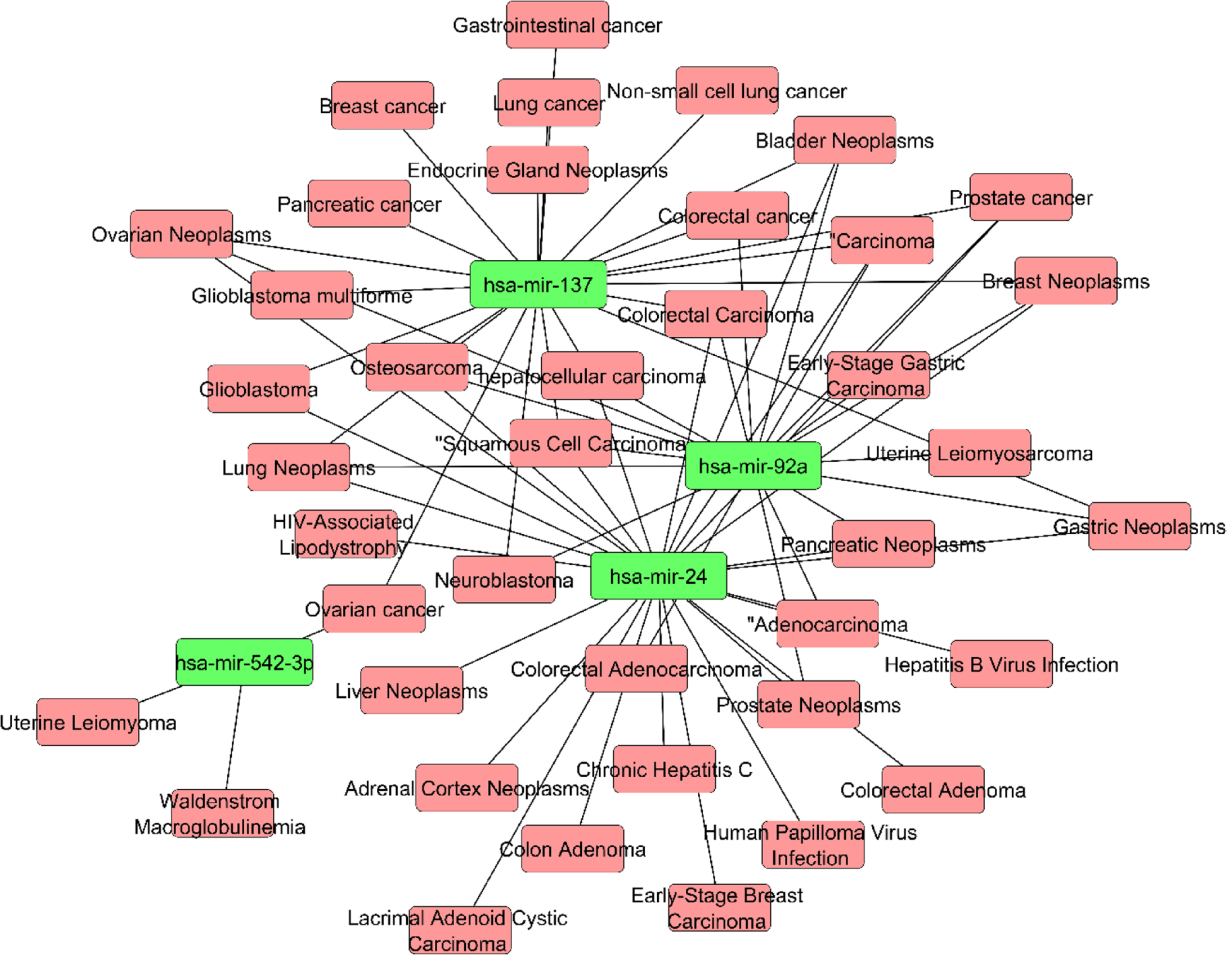
miRNA disease association network showing the interconnection between the miRNA (green color) and disease (red color)

Transcription factors (TFs) and microRNAs (miRNAs) are important regulators of gene expression [63]. In CC, miRNAs and TFs may perform a dual regulatory role. We built a complete particular TF-miRNA co-regulatory network by merging predicted and empirically confirmed TF and miRNA targets after gathering hub genes from the PPI network. Using hub genes, the RegNetwork repository was utilized to build a TF-miRNA co-regulatory network. There are 112 nodes and 136 edges in the TF-miRNA co-regulatory network, including 73 TF candidates, 8 hub nodes, and 31 miRNA candidate nodes (Fig. 8). In addition, 31 miRNA were analyzed to detect the cancer disease connectivity (Fig. 9). In the figure, hsa-mir-137, hsa-mir-92a, and hsa-mir-542-3p show the high connectivity with the disease including breast cancer, ovarian cancer, pancreatic cancer, glioblastoma, non-small cell lung cancer, and bladder neoplasms.

### Survival analysis

**Fig. 10:**
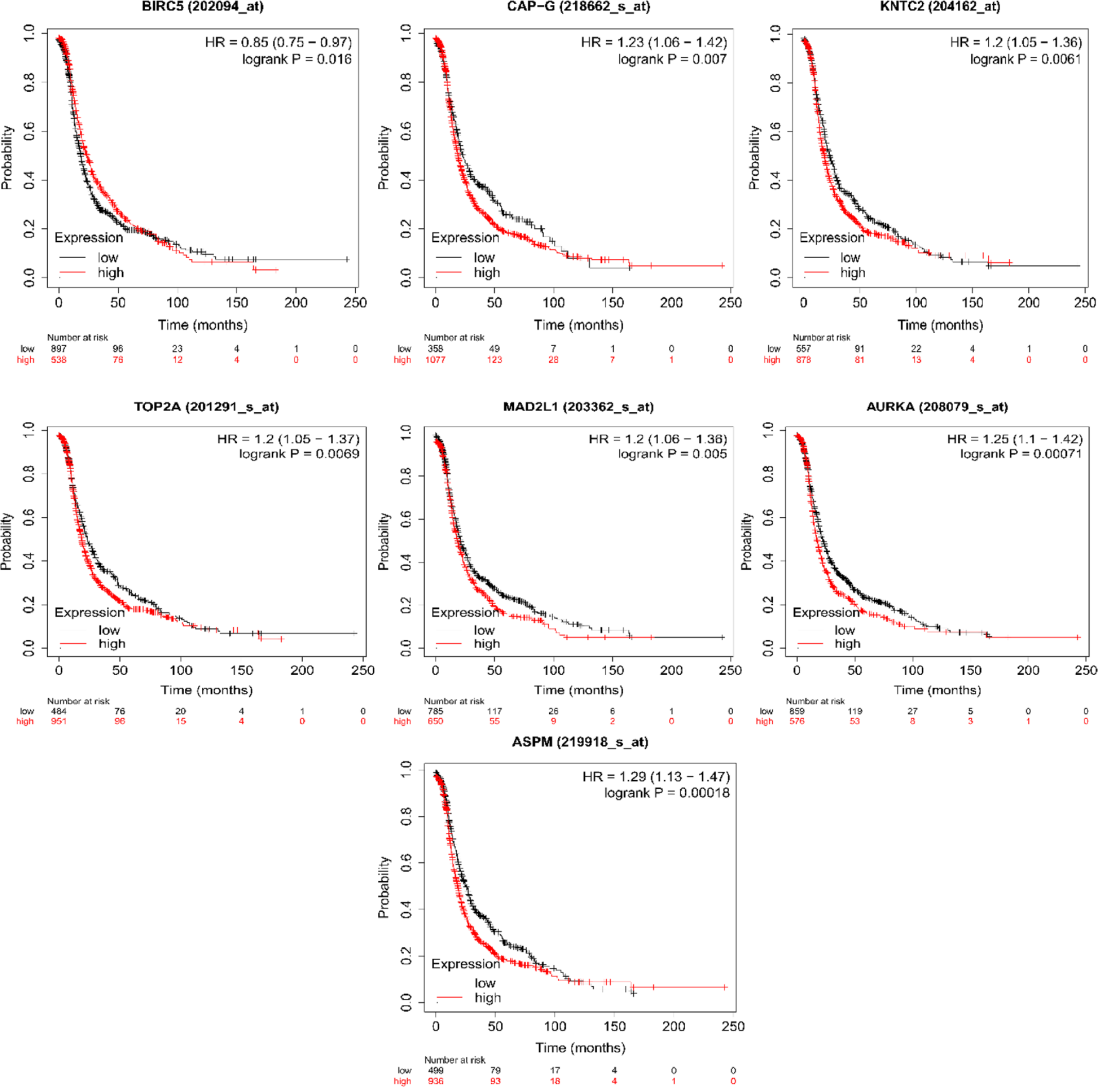
This figure showed the survival rating of patient-associated CC with the common proteins. The survival plot has taken with the logrank *p* < 0.05. The survival plot is made with two axis named probability and time (months). All the hub proteins were analyzed for survival rating, and seven proteins including BIRC5, NCAPG/CAPG, TOP2A, MAD2L1, AURKA, ASPM, and NDC80/KNTC2 perform less than 0.05

Following the analysis, were picked are the most critical genes of CC with the cutoff p-value <= 0.05. The PL estimator had been used to achieve the survival curves of the most relevant genes in contrast with altered and regular populations. All the hub genes were analyzed for survival rating, and seven proteins including BIRC5, NCAPG/CAPG, TOP2A, MAD2L1, AURKA, ASPM, and NDC80/KNTC2 perform logrank P less than 0.05 (Fig. 10). CAPG could play a significant role in the survival of breast cancer [64], bladder cancer [65], as well as ovarian cancer [66], and many more. This finding reports for the first time that CAPG may play a significant role in the survival of cervical cancer. Although MAD2L1 [67], TOP2A [68], AURKA [69], and ASPM [68] were reported in various research findings, they may play a remarkable role in the survival of cervical cancer. By contrast, NDC80 and CAPG are a novel targets in the survival of cervical cancer.

## Discussion

CC is the fourth most frequent cancer in women worldwide and the fourth leading cause of cancer death. According to estimations, CC claimed the lives of 342,000 individuals in 2020. Around 8% of all cancer diagnoses and deaths are caused by this condition. We used a network-based technique to look at the patterns of gene expression in three microarray datasets of CC patients and found molecular targets that could be employed as cancer biomarkers. It could also disclose crucial information about their impact on the progression of illnesses or disorders. In the fields of biomedical and computational biology, expression profiling utilizing high-throughput microarray datasets has proven to be a helpful resource for identifying biomarker candidates for a variety of diseases [70]. According to the CC transcriptomics analysis, the common 315 DEGs have comparable expression in three datasets. To obtain insight into the etiology of CC, the biological relevance of 315 frequently DEGs was examined utilizing gene ontology and pathway analysis techniques based on *p*-values.

The Gene Ontology (GO) is a gene regulation framework based on a generic conceptual perspective that makes it easier to comprehend genes and their interactions. Evolution achieved this over time by accumulating biological knowledge about gene functions and regulation in a variety of ontological domains [71]. For three types of GO analysis, the GO database was employed as an annotation source: BP (molecular activities), CC (gene controls function), and MF (activities at the molecular level) [72]. The biological process (34 genes) and DNA metabolic process (34 genes) are the most important, followed by transcription control (43 genes). Many cellular metabolic activities involve deoxyribonucleic acid. This is one of the two primary types of nucleic acid, and it is made up of one or two strands of connected deoxyribonucleotides [73]. The method by which a cell controls the translation of DNA to RNA (transcription) to regulate gene activity is called transcriptional regulation. Changing the quantity of copies of RNA produced and manipulating when the gene is transcribed are two examples of how a single gene can be managed [74].

Most of the gene promoters in invertebrates have a CpG island with several CpG sites [75]. A gene is silenced when several of its promoter CpG sites are methylation [76]. However, transcriptional silencing may have a bigger role in cancer development than mutation. For the cellular component function, the nuclear chromosome part (33 genes), microtubule-organizing center (22 genes), and chromatin (18 genes) are significant. According to the molecular function, top GO terms peptidase activity, acting on L-amino acid peptides (12 genes), kinase binding (19 genes), and DNA binding (32 genes), are significantly associated.

Pathway analysis [77] is the most effective method for reflecting an organism’s behavior via internal changes. The pathways of the most prevalent DEGs were culled from three separate databases: KEGG, Reactome, and WikiPathways. Cell cycle, DNA replication, cellular senescence, the p53 signaling route, drug metabolism, prostate cancer, human T-cell leukemia virus 1 infection, chemical carcinogenesis, and cancer pathways are the top ten KEGG pathways. Cell cycle, mitotic, mitotic G1-G1/S phases, S phase, G1/S transition, cell cycle checkpoints, M phase, generic transcription pathway, and metabolism are all heavily connected with common DEGs, according to Reactome’s pathways. The most prevalent DEGs connected with WikiPathways route include retinoblastoma gene in cancer, G1 to S cell cycle control, cell cycle, DNA IR-damage and cellular response via ATR, miRNA regulation of DNA damage response, vitamin D receptor pathway, and integrated breast cancer pathway.

Using common DEGs, a PPI network had been created to understand the biological characteristics in-depth and explore disease biomarkers. Depending on the topological measure (degree), 10 hub genes have been traced from the PPI network, which might be a therapeutic target or biomarker. The top 10 hub genes including CDK1, CCNB1, CDC20, TOP2A, MAD2L1, NDC80, AURKA, ASPM, NCAPG, and BIRC5 showed high degree value. In CC, cyclin-dependent kinase 1 (CDK1) has been observed before. CDK1 is a highly preserved protein that works as a serine kinase/threonine. With over 70 regulatory objectives, it plays a key role in controlling the cell cycle. CDK1 phosphorylates directly a number of target substrates for controlling the transcription and progression of cells in response to different stimuli [78]. Studies have demonstrated that CDKs and their modulators are aberrantly activated in several malignancies. CDK dysregulation induces the growth of aberrant cells and genomic instability [79]. Indeed, all human malignancies are affected by the D-cyclin-cdk4/6-INK4-Rb pathway [80]. Research from in vitro and in vivo shows that a variety of malignancies including cervical, colon, and breast cancer have substantial anticancer effects using CDK inhibitors [81, 82].

The transcriptional and posttranscriptional regulators of the hub DEGs were discovered using the miRNA-TF co-regulatory network. TFs govern transcription ratios, whereas miRNAs play a significant role in gene posttranscriptional regulation and RNA silence. The role of transcription factors (TFs) and microRNAs (miRNAs) in the progression of disease is crucial. Thus, the connections between the common DEGs, TFs, and miRNAs are shown in our research. The network builds on 73 TF candidates, 8 hub nodes, and 31 miRNA candidates. From the miRNA candidate, 4 candidates (hsa-mir-137, hsa-mir-92a, hsa-mir-24, and hsa-mir-542-3p) showed significant association with various types of cancer. miR-137 is integrated into CpG island (a high-frequency genomic area that contains CpG dinucleotide) and has been found in several types of cancer, such as colorectal, gastric, breast-and-squamous cells, and head and neck to have often been silenced by the promoter of hypermethylation [83–85]. MiR-137 is inhibited epigenetically in colorectal adenomatous cells in the same way as it is suppressed in colorectal cancer tissue, showing that miRNA methylation occurs early in colorectal cancer [86]. There are various studies which showed that hsa-mir-92a miRNA significantly connected with CC [87], colorectal cancer [88], small cell lung cancer [89], breast cancer [90], etc. According to some previous studies, hsa-mir-24 miRNA might play a significant role in lung cancer [91], breast cancer [92], prostate cancer [93], and colorectal cancer [94].

Gene-disease association network was analyzed to reveal the gene connectivity with the disease. The outcomes of this analysis showed liver carcinoma, lung carcinoma, adenocarcinoma, non-small cell lung carcinoma, renal cell carcinoma, colorectal neoplasms, prostatic neoplasms, neuroblastoma, kidney neoplasms, adrenocortical carcinoma, etc. are significantly connected with the hub DEGs. In individuals with CC, the history of radiation was an independent risk factor for second primary lung cancer [95]. A study indicated that 4.33% of CC patients develop lung metastasis [96], which is consistent with prior research [97, 98].

Another biggest exploration of this study is drug compounds finding for cancer. This analysis showed many compounds are connected with cancer. From them, LUCANTHONE CTD 00006227 and paclitaxel CTD 00007144 targets are significantly associated with CC. “LUCANTHONE” is the new target for CC. In previous, some studies have announced “LUCANTHONE” reduces cancer cell survival regardless of p53 status; autophagy suppression may be a more important contribution to the lucanthone mode of action that impacts DNA repair. Another drug compound target “paclitaxel” was studied as an anticancer medication in some previous studies [58–60]. The authors in these studies wrote, “paclitaxel” may play a significant role in CC as well as ovarian cancer, head and neck cancer, esophageal cancer, breast cancer, and lung cancer. Paclitaxel has a key role in the care of advanced/metastatic illness in cervical cancer, according to the European Society for Medical Oncology (ESMO) clinical practice guideline issued in 2017 [59]. Paclitaxel and cisplatin in combination with bevacizumab are regarded as the optimal first-line regimens for metastatic or recurrent cervical cancer due to the appropriate balance of effectiveness and safety characteristics. Paclitaxel with carboplatin may be an alternate choice for individuals who are not candidates for cisplatin, particularly those with impaired renal function [99]. Paclitaxel has been the first microtubule-stabilizing agent identified and considered as a significant part of the standard chemotherapy regimens for treating cervical cancer. In particular, the combination of paclitaxel with platinum-derived drugs has represented and represents today the cornerstone of advanced cervical cancer therapy, in particular for women with recurrent and persistent disease [59].

## Conclusion

In this study, three datasets of CC were analyzed and compared to identify the important DEGs. A total of 315 common DEGs have collected for further analysis. Using common DEGs were used the functional analysis to figure out important GO terms and pathways using KEGG, Reactome, and WikiPathways database. Also, we developed a PPI network and identified significant hub DEGs including CDK1, CCNB1, CDC20, TOP2A, MAD2L1, NDC80, AURKA, ASPM, NCAPG, and BIRC5 that are the main therapeutic targets for the CC. And gene disease association and TF-miRNA co-regulatory network also demonstrated to identify the drag target miRNA. The analysis of the drug compounds showed LUCANTHONE CTD 00006227 and paclitaxel CTD 00007144 are the most associated with the hub DEGs. This network-based study may play a significant role in future treatment or medicine development for the patient with CC.

## Data Availability

The datasets used and/or analyzed during the current study are available from the corresponding author on reasonable request.
